# Beekeeping in Europe facing climate change: A mixed methods study on perceived impacts and the need to adapt according to stakeholders and beekeepers

**DOI:** 10.1016/j.scitotenv.2023.164255

**Published:** 2023-08-25

**Authors:** Marie Van Espen, James H. Williams, Fátima Alves, Yung Hung, Dirk C. de Graaf, Wim Verbeke

**Affiliations:** a Ghent University, Department of Agricultural Economics, Coupure links 653, B-9000 Gent, Belgium; b Aarhus University, Department of Ecoscience (ECOS), C.F. Møllers Allé 4-8, 8000 Aarhus C, Denmark; c University of Coimbra, Centre for Functional Ecology, Science for People and the Planet, TERRA Associate Laboratory, Calçada Martins de Freitas, 3000-456 Coimbra, Portugal; d Universidade Aberta, Lisbon, Portugal; e Ghent University, Department of Biochemistry and Microbiology, Krijgslaan 281 S2, B-9000 Ghent, Belgium

**Keywords:** Apiculture, Climate adaptation, *Apis mellifera*, Perception, Pollinators decline

## Abstract

The beekeeping sector is suffering from the detrimental effects of climate change, both directly and indirectly. Despite numerous studies conducted on this subject, large-scale research incorporating stakeholders' and beekeepers' perspectives has remained elusive. This study aims to bridge this gap by assessing the extent to which stakeholders involved in the European beekeeping sector and European beekeepers perceive and experience the impacts of climate change on their operations, and whether they had to adapt their practices accordingly. To this end, a mixed-methods study including in-depth stakeholder interviews (*n* = 41) and a pan-European beekeeper survey (*n* = 844) was completed within the frame of the EU-funded H2020-project B-GOOD. The development of the beekeeper survey was informed by insights from literature and the stakeholder interviews. The results highlighted significant regional disparities in the perceived impacts of climate change, with beekeepers in Southern European regions expressing more negative outlooks, while Northern European beekeepers reported more favourable experiences. Furthermore, survey analysis revealed beekeepers who were classified as ‘heavily impacted’ by climate change. These beekeepers reported lower average honey yields, higher colony winter loss rates and a stronger perceived contribution of honey bees to pollination and biodiversity, underscoring climate change's detrimental impacts on the beekeeping sector. Multinomial logistic regression revealed determinants of the likelihood of beekeepers being classified as ‘heavily impacted’ by climate change. This analysis indicates that Southern European beekeepers experienced a 10-fold likelihood of being classified as heavily impacted by climate change compared to Northern European beekeepers. Other significant factors distinguishing ‘winners’ and ‘losers’ were self-reported level of professionalism as a beekeeper (ranging from pure hobbyist to fully professional, Odds Ratio (OR) = 1.31), number of years active in beekeeping (OR = 1.02), availability of floral resources throughout the bee season (OR = 0.78), beehives located in a forested environment (OR = 1.34), and the presence of local policy measures addressing climate change-related challenges (OR = 0.76).

## Highlights

• Climate change (CC) is clearly perceived to be impacting beekeeping in Europe.
• CC impacts are likely to create winners and losers within the beekeeping sector.
• Major CC impacts concern local weather conditions and food resource availability.
• CC impacts are associated with lower honey yield and higher colony winter loss.
• Southern, professional and ‘forest’ beekeeping boost chance of being impacted by CC.

## Graphical abstract

**Figure:**
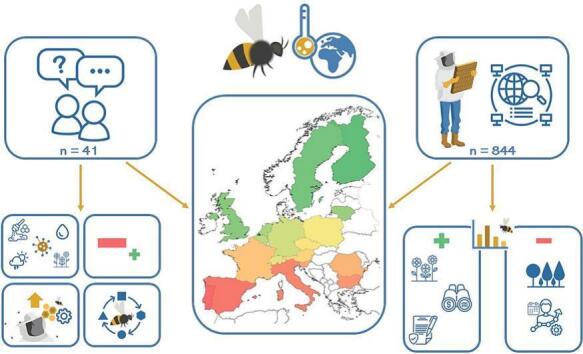

## 1 Introduction

“What used to be normal years in the past, are now exceptionally good years, and what used to be exceptionally bad years, are now normal years.” – *Apiary product quality inspector, referring to the evolution of honey yields in Crete, Greece*

*(personal communication, September 2022)*

“Quand je compare la situation d'aujourd'hui avec celle de l'époque où j'aidais mon père en tant qu'apiculteur, c'est une différence de jour et de nuit.” [Translation: “When I compare the situation today with when I was helping my father as a beekeeper, it's a night and day difference”] – *Beekeeper, selling apicultural products at the farmers' market Marché Edgar-Quinet in Paris, France*

*(personal communication, October 2022*)

The two quotes above paint a rather bleak picture about the perceived and experienced impact of climate change on the beekeeping sector. The agricultural sector in general has been suffering the consequences of more irregular seasons and extreme weather events, as well as rising temperatures (Intergovernmental Panel on Climate Change [IPCC], 2022). These climatic changes are causing shifts in the blooming periods and habitats of several plant species, also triggering spatial and temporal mismatches between pollinators and their floral food resources (Bartomeus et al., 2011; Memmott et al., 2007; Phillips et al., 2018). Furthermore, the changing environmental conditions brought about by climate change are opportune for pests and diseases to spread outside of their usual ranges (Abou-Sharaa et al., 2021; Cornelissen et al., 2019; Giliba et al., 2020). Some regions are more prone to negative climate effects than others (Flores et al., 2019), indicating a geographical component to the impact of climate change on agriculture and apiculture alike (IPCC, 2022). To gain insights into the effects of climate change on beekeeping in Europe, this study investigated how actors in the European beekeeping sector perceive and experience the impacts of climate change related to beekeeping, through interviews with stakeholders and a large-scale beekeeper survey.

In their 2022 report, the IPCC asserts that intensifying climate change has already pushed millions of people into acute food insecurity and predicts that agrifood systems will also be affected in the longer term due to, among others, drought stress, altered seasonality, heavy rain events and increasing mean temperatures (IPCC, 2022). These are examples of direct effects of climate change on agricultural systems, whereas indirect effects are felt through the climate change-induced fluctuations in other (plant and animal) species that are key for (agricultural) biodiversity, such as pests and their natural enemies, soil organisms, and pollinators (FAO, 2015, FAO, 2019). Indeed, more than a third of global crop production depends on insects for pollination (Powney et al., 2019), with bees being key pollinators and honey bees (*Apis mellifera*) being commonly used for agri- and horticultural crop pollination (Potts et al., 2016) besides the production of apiary products such as honey, pollen, royal jelly, and beeswax.

Climate change can affect the beekeeping sector in a variety of ways, and a common impact is the altered availability of food resources. Rising temperatures can cause disruptions in the flowering seasons of many floral species, by either shifting the starting date or by shortening/lengthening the blooming period (Bartomeus et al., 2011; Langowska et al., 2017; Medina-Cuéllar et al., 2018). Since the foraging activity of honey and wild bees is also regulated by temperature, these shifts may cause temporal mismatches between the pollinators' activities and their floral food resources (Memmott et al., 2007). Furthermore, certain areas may become unsuitable for certain types of plants, leading to spatial mismatches between plants and (honey) bees (Castellanos-Potenciano et al., 2017). While honey bees are foraging generalists - i.e., they feed on a wide range of floral resources (Valido et al., 2019) -, their survival could be threatened by spatial and temporal mismatches as well. This is because the quality and quantity, but more importantly, the diversity of their pollen supply greatly influences their health and survival (Donkersley et al., 2014; Montoya-Pfeiffer et al., 2021). In addition, more extreme weather events – be it severe droughts or heavy rainfall – reduce the overall floral abundance, decrease pollen and nectar production and availability, and/or deplete the available pollen's nutritional quality (Le Conte and Navajas, 2008; Newman et al., 2021; Phillips et al., 2018), exacerbating this problem. Moreover, some pollinators – including honey and wild bees - may experience difficulties in adapting to these changing environments, e.g., due to the fragmentation of landscapes in the North Temperate Zone (in which most of Europe is located) (Vasiliev and Greenwood, 2021). Consequently, certain types of honey are at risk of disappearing as a result, such as acacia honey in specific regions (Novelli et al., 2021).

Certainly, environmental changes have major consequences for pollinators in general and bees in particular. In addition to reduced food resource availability, for managed honey bee species, climate change is also unlocking previously uninvaded (European) areas to various pests and diseases, such as the greater wax moth (Hosni et al., 2022), the small and large hive beetle (Abou-Sharaa et al., 2021; Cornelissen et al., 2019), and the Asian hornet (Requier et al., 2019). Moreover, pests already plaguing honey bees worldwide, such as *Varroa destructor*, can also benefit from increasing temperatures, enabling them to survive milder winters (Vercelli et al., 2021). Unfortunately, the rate of pathogen infestation is likely to increase as beekeepers resort to seasonal movement of colonies (so-called ‘transhumance’) in response to declining local food resources (Gajardo-Rojas et al., 2022). Additionally, rising temperatures increase the stress experienced by honey bee hives (Yildiz and Ôzilgen, 2019), which further heightens the bees' susceptibility to pathogens (Graystock et al., 2016).What is more, hibernation and brood rearing activity in honey bee hives is profoundly sensitive to changes in ambient temperatures, causing hives to end their hibernation too early, or to forego their hibernation period altogether (Nürnberger et al., 2018).

Furthermore, climatic changes may not affect all bees and beekeepers equally, as the climate change-induced thermo-pluvial variations differ depending on geographical location (IPCC, 2022). In Europe, the Mediterranean region seems to have become particularly vulnerable to extreme temperatures and droughts, with climatic conditions exceeding the optimal temperature for nectar secretion for many floral species (Flores et al., 2019; Gérard et al., 2020; Novelli et al., 2021). By contrast, several studies in Northern European regions (e.g., Poland and the United Kingdom) have revealed that rising temperatures extend the beekeeping season there, exposing local bees to new flows of nectar (Langowska et al., 2017).

A systematic review by Decourtye et al. (2019) of the literature on ‘bee threats’ revealed that, as of 2019, studies on climate change only constituted 3.2 % of recent research, with most of the climate-related research having been conducted from a natural sciences perspective. However, it is equally important to look at climate change effects through the lens of stakeholders and the beekeepers themselves, as they have specialised knowledge concerning their bees, their surroundings, and the local climate, which in turn shapes their perception of climate change and may affect their adoption of adaptation strategies (Gallardo-López et al., 2021). Therefore, this paper presents the first pan-European study that draws upon the expertise and views of stakeholders from distinct parts of the beekeeping sector, who participated in interviews, and a survey completed by hundreds of beekeepers, inquiring about their perceptions of and experiences with climate change. More specifically, this research focused on two main topics: (I) the extent to which European beekeepers perceive and experienced various (positive or negative) impacts of climate change on their beekeeping operations, and (II) whether they have had to change or adapt their beekeeping practices in order to deal with these impacts. While there have been some studies about climate change and sustainability in the beekeeping sector involving interviews and surveys with stakeholders from the apicultural sector and beekeepers in recent years, none have been quite as large-scale as the present study performed within the frame of the EU-H2020-funded project B-GOOD (de Graaf et al., 2022). Previous similar studies mostly included only one of two respondent groups – either stakeholders or beekeepers, had a limited amount of study participants (Gajardo-Rojas et al., 2022; Gallardo-López et al., 2021; Newman et al., 2021), focused on the situation in a very specific region or single country (Novelli et al., 2021; Vercelli et al., 2021), did not elaborate explicitly on the topic of climate change (Buchori et al., 2022; El Agrebi et al., 2021), or focused on an entirely different topic altogether, such as a sustainability assessment framework for the beekeeping sector (Kouchner et al., 2019) or management practices to reduce winter colony loss (Steinhauer et al., 2021).

## 2 Materials and methods

Primary data were collected through a mixed-methods research approach including a qualitative exploratory study with stakeholders followed by a quantitative descriptive study with beekeepers. Firstly, stakeholders (*n* = 41) involved in the European beekeeping sector were interviewed, with questions used to explore their views and opinions regarding the impact of climate change on beekeeping. Secondly, European beekeepers (*n* = 844) were surveyed using an online questionnaire to assess their perceptions and experiences of climate change impacts on their beekeeping activities.

### 2.1 Stakeholder in-depth interviews

#### 2.1.1 Topic guide

The stakeholder interview topic guide contained three main sections, consecutively addressing stakeholder views on the business- and macro-environment facing the European beekeeping sector, the status of honey bee colony health, and current and future beekeeping practices and related challenges. This third section contained two open-ended questions specifically addressing the topic of climate change impacting beekeeping practices and the distinct challenges it posed. The open-ended probing questions were: ‘To what extent do you think climate change has had an impact on beekeeping, and how so?’ and ‘What impact do you think climate change will have on beekeeping in the future?’. Both questions were framed explicitly within the European context. The topic of climate change was spontaneously raised by half of the interviewees earlier on during the interview, e.g., as an external environmental factor influencing the beekeeping sector (*n* = 16) or as a specific factor impacting honey bee colony health (*n* = 5), whereupon the topic was discussed in depth using the questions above and not revisited at a later stage during the interview.

#### 2.1.2 Participants and recruitment procedure

The in-depth stakeholder interviews were completed during January–March 2020. Individual stakeholders were recruited through a mix of non-probability snowball and convenience sampling (Johnson and Christensen, 2014). Initial contacts were selected among members of the EU Bee Partnership, the International Honey Commission, and the Food and Agricultural Organization of the United Nations (FAO), representing international networks of actors involved in the beekeeping sector. Contacted stakeholders were asked to provide up to three additional potential contacts for further interviews. This procedure was continued until saturation was reached in the diversity of actors and it was deemed sufficient insights were gained on the questioned topics. All stakeholder interviews were completed before Europe was declared the epicentre of the COVID-19 pandemic.

The interviewed stakeholders were classified based on their primary activity related to the beekeeping sector as scientists (*n* = 9), service providers (*n* = 7) - including veterinary and extension services -, policy makers (*n* = 5), representatives of non-governmental organisations (NGO) (n = 5), actors involved in the agricultural and horticultural sector (n = 5), actors involved in product quality inspection and control (n = 5), and representatives of beekeeper associations (n = 5). Most interviewees represented multiple functions and could therefore fit in several categories, e.g., scientists or NGO- representatives who were also involved in quality inspection, service provision or active in a beekeeper association. The analysis is attentive to the diversity of views and opinions among the different stakeholder groups and seeks to outline the general picture emerging from the breadth of interviews and provide input for the quantitative beekeeper survey, which is consistent with the aims of qualitative exploratory research.

#### 2.1.3 Content analysis

The stakeholder interviews were conducted in English, apart from a few exceptions in French (*n* = 2) or Portuguese (n = 2). All interviews were audio-recorded, transcribed verbatim using NVivo's on-line transcription service and then manually checked, and translated into English when needed. Qualitative content analysis was done using deductive coding in NVivo. Recurring topics were identified using coding matrices to determine the various domains in which climate change affects the apicultural sector and to serve as input for the beekeeper survey. Verbatim statements were selected to illustrate specific views and opinions related to the study topic.

#### 2.1.4 Ethics approval

Ethics approval for the stakeholder interviews was granted by the Ethics Committee of Ghent University's Faculty of Psychology and Educational Sciences (ref. nr. 2019/122 – January 2020).

### 2.2 Beekeeper survey

#### 2.2.1 Questionnaire and measurement scales

A quantitative descriptive survey was implemented for collecting data from EU beekeepers about a diversity of topics including socioeconomic characteristics of their beekeeping operation, personal attitudes, and management practices and decisions in relation to beekeeping, production efficiency, honey bee colony health and its monitoring, environmental quality, and interest in digital technologies for beekeeping. The study's participant information sheet referred to ‘a study on the socioeconomics of beekeeping as part of the project B-GOOD that aims to pave the way to healthy and sustainable beekeeping in Europe.’ Questions probing for the perceived and experienced impact of climate change were included in the questionnaire's section dealing with environmental quality. The content of these questions was primarily informed by the insights obtained from the stakeholder interviews, complemented by a limited scoping literature review using the following search terms: [(climate change) AND ((honey bee* OR honeybee* OR *Apis mellifera*) OR (beekeeping OR apiculture))].

Participants were first asked to indicate their level of agreement with the statement ‘Climate change has forced me to change my beekeeping practices’ using a five-point Likert (interval) scale ranging from ‘strongly disagree’ to ‘strongly agree’. Next, they were exposed to the statement ‘According to my personal experience, climate change has a … impact on my beekeeping activities’ and again asked to record their response on a five-point interval scale ranging from ‘very negative’ to ‘very positive’. Finally, beekeepers were asked to indicate to what extent (positive or negative) they considered climate change impacting their beekeeping in relation to four natural environmental factors (food resource availability, water availability, local weather conditions, and natural disasters like fires or flooding) and five factors relating to honey bee colony behaviour and performance (length of the bee season, disease infestation, honey yield, colony survival, and swarming behaviour) (see Supplementary Material for an overview of question wording and response options).

A myriad of socioeconomic data was collected, including country of residence, age, gender, number of years active as a beekeeper, number of beehives in 2021, hobbyist vs. professional beekeeper (based on the size of their beekeeping operation and based on their beekeeping skills), rural vs. urban location, their colonies' natural environment (e.g., forestry, presence of floral resources throughout the bee season), total quantity of honey produced in 2021 (kg), average beehive winter loss percentage over the past five years, and the beekeeper's assessment of the most recent bee season (2021) in terms of honey production and economic performance. The survey questionnaire was developed and pre-tested in English with the collaboration of those members of the research project consortium who are also beekeepers. The master English version of the questionnaire and all related informed consent literature were translated into 11 additional languages, further pre-tested and checked for linguistic equivalence. All language versions were web-programmed in the survey software Qualtrics for online administration.

#### 2.2.2 Data collection procedure

Beekeepers could participate in the survey during October 2021–January 2022. Access to the survey was provided through a dedicated website where study participants could select their native (or preferred) language version of the questionnaire. Participant recruitment was performed through distributing the survey's web link through national beekeeping associations who posted the survey invitation on their websites, newsletters, and social media posts. To aim for as many answers from beekeepers as possible, additional recruitment efforts were done by the researchers through beekeeper contacts of the involved partner research institutes, and (social and mass) media and communication contacts of consortium partners in their national and regional beekeeping communities, though only in countries or regions where national or regional beekeeping associations had agreed previously to participate. Duplicate submissions were prevented through the use of appropriate software (Qualtrics). The study qualifies as a self-administered internet survey using a non-random participant self-selection sampling method, as described by van der Zee et al. (2013). This data collection method has several benefits (e.g., related to reach, speed, cost, and data transfer) as well as some limitations, which will be discussed in the ‘Discussion section’ in relation to our study.

#### 2.2.3 Statistical analysis

Participant's socioeconomic characteristics were summarised using descriptive statistics. Differences between groups (based on e.g., age, gender, degree of professionalism) in their perceptions and experiences of climate change were assessed using the Mann-Whitney two-sample statistic in the case of a binary variable of interest, and a more general Kruskal-Wallis test if there were more than two response categories, as both the ‘climate change forced me to change practices’ and ‘perceived impact of climate change’ variable were not normally distributed. A new binary variable that indicates whether a beekeeper had been heavily impacted by climate change was created by grouping those participants that (strongly) agreed with the statement that ‘climate change forced them to change their beekeeping practices’ and indicated they experienced ‘a (very) negative impact of climate change on their beekeeping activities.’ Next, bivariate associations between this new variable (further referred to as ‘heavily impacted’) and socioeconomic characteristics were tested through cross-tabulation and chi-square statistics. Finally, a multinomial logistic regression model was estimated with this newly constructed binary ‘heavily impacted’ variable as the dependent variable and selected explanatory variables. Using the estimates of this model, probabilities of classifying as ‘heavily impacted’ by climate change were simulated for different EU regions and across the range from hobbyist to professional beekeepers. The choice for simulating probabilities across regions and degree of professionalism was informed by their statistical significance and empirical interest. Statistical analyses were performed using StataCorp Stata 17 and IBM SPSS Statistics 25.0. Probability simulations were done with Microsoft Excel 2019.

#### 2.2.4 Ethics approval

Ethics approval for the beekeeper survey was granted by the UZ Gent/UGent Medical Ethics Committee (ref. nr. BC-10610 – August 2021).

## 3 Results

### 3.1 Stakeholder in-depth interviews

#### 3.1.1 Stakeholder characteristics

Of the 41 interviewees, 31 identified as male, 10 as female, and their age ranged from 34 to 79 years. They all lived and worked in Europe, representing 10 different nationalities, with France, Germany and Belgium topping the list. This reflects the fact that most interviewees had EU-level working positions, often centred around EU institutions. In total, 31 of the interviewed stakeholders ended up discussing climate change, either as an external environmental factor affecting beekeeping or in the section on current and future challenges facing the beekeeping sector. There were no groups of stakeholders (based on age, gender, nationality, or stakeholder category) that discussed climate change significantly more or less than others. The main reason why some stakeholders did not discuss climate change was not feeling sufficiently informed or having the expertise to do so.

#### 3.1.2 Content analysis

There are four overarching themes that emerged from the content analysis of the interviews. First, many interviewees explicitly stated which impacts of climate change on beekeeping activities they perceived or expected. The top part of Table 1 provides an overview of the several types of impacts, and how many stakeholders mentioned a specific perceived impact during their interview. According to those interviewed, the main impacts of climate change on the beekeeping sector are or will be in relation to food resource availability, changing local weather conditions, the length of the bee season, and disease infestation. These impacts were not perceived to occur in isolation but are clearly interlinked, as illustrated by this excerpt of one of the interviews:

“Bees are regulated by temperature, but this year we had bees flying in January. This is rather ridiculous, because bee activity and plant flowering should be correlated. What we see now is that biological calendars are no longer synchronised. […] Summers are becoming hotter and drier, which means that less flowers are available. […] Pathogens and parasites are also not freezing to death anymore because of the lamentable winters we are having. You need a good winter so that parasite populations have to start from scratch in the new year. Now, however, a much higher threshold remains, which causes more and easier infestation, and will definitely have drawbacks for bee health.” –

*Scientist, 44 years old, the Netherlands*

**Table 1:** Overview of different types of climate change impacts mentioned by stakeholders (SH), and stakeholders' inclination towards climate change effects; n = number of stakeholders (out of 31^a^).

Various types of climate change impacts mentioned by stakeholders	
Impact type	n
Food resource availability	15
Local weather conditions	14
Length of the bee season	11
Disease infestation	9
Honey yield	8
Colony survival	7
Natural disasters	3
Water availability	2
Stakeholders' inclination towards climate change effects^a^	
Climate change is affecting the beekeeping sector	n
SH mentions only positive effects of climate change	0
SH mentions only negative effects of climate change	16
SH mentions both positive and negative effects of climate change	10
SH mentions climate change effects without explicitly defining if they are positive or negative	2
Climate change is not affecting the beekeeping sector	
SH states that climate change is not affecting the beekeeping sector	2
SH does not know if climate change is affecting the beekeeping sector	1

a Out of the total number of 41 interviewed stakeholders, 10 did not expand on the topic of climate change.

Other areas in which the interviewees perceive or expect an impact from climate change include the honey yield, colony winter survival, the occurrence of natural disasters, and water availability.

Secondly, although negatively perceived and expected impacts from climate change prevailed during the stakeholder interviews, climate change was not unequivocally seen as having negative impacts only for the beekeeping sector. The bottom part of Table 1 shows that – while 16 stakeholders indeed suggest negative impacts of climate change only – ten stakeholders mentioned both positive and negative effects. Yet, none of the stakeholders who discussed climate change mentioned positive impacts only, as indicated by the following quote.

“I think it's probably not black and white. Climate change can bring disadvantages, threats, as you would say in a SWOT, but it can also bring opportunities.” –

*Horticultural actor, 55 years old, Germany*

Additionally, two stakeholders did not indicate a clear direction of the perceived impacts they mentioned, and three did not believe climate change was or will be affecting the beekeeping sector. There was consensus among stakeholders that whether climate change has or will have negative or positive impacts on the beekeeping sector, depends largely on the geographic location or European region, with negative impacts expected mostly in Southern European regions, and positive impacts rather in Northern European regions.

“Climate change is leading to more irregular harvests, and more so in certain areas of Europe than in others. It is an opportunity in certain more Northern countries where they will have longer harvesting periods, so in some areas it is positive and in others negative.” –

*NGO representative, 39 years old, Belgium*

“Yes, the climate has changed, but for me not necessarily negatively. [In Sweden], we will be able to produce more honey if the temperature increases.” –

*NGO representative, 78 years old, Belgium*

“Yeah, [climate change has had an impact] especially in Southern Europe because it is becoming dryer and the seasons for beekeeping are changing. Because of the drought, bees have less food available in the summer.” –

*Policy maker, 52 years old, France*

Thirdly, stakeholders indicated that climate change is imposing additional challenges on beekeeping in general and is forcing many beekeepers to change their beekeeping practices. For instance, some interviewees mentioned that beekeeping may become more challenging, more time-consuming, and will require additional skills and efforts from beekeepers. For example, they referred to the likelihood that honey bee colonies will need to be monitored all year round and checked more frequently, particularly with regard to food stocks, newly emerging pests and disease infestation.

“Beekeepers really have to watch out because their bees can burn through their winter food stock faster than anticipated. They can't just put their bees in the hive, winter them in and look at them again four months later. Those times are gone. Beekeepers have to be there more often. There will be more gaps in nectar availability throughout the year. That will have an impact on beekeeping and what beekeepers can do and cannot do anymore with their bees.” –

*Scientist, 44 years old, the Netherlands*

“I would say that climate change will be a selection criterion for beekeepers [rather than for bees]: those who do not have enough knowledge will run out of bees and give up. Those who can adapt their colonies and hives to the real situation, will find ways to overcome that.” –

*Policy maker, 47 years old, Portugal*

Lastly, some stakeholders were not overly pessimistic, and several (*n* = 7) mentioned that honey bees – as a species – are quite resilient, flexible, and adaptable to different environments. Stakeholders were generally convinced that the honey bee as a species would manage to cope with changing environments and survive, although some expected that climate change could lead to some shifts in the dominance of subspecies because of changes in the regional suitability of natural environments. It was apparent from interviewee responses that climate change may not be directly affecting the honey bees themselves, but mostly impacted indirectly through the changed availability of habitats and (food and water) resources and the increased prevalence of pests and diseases. These environmental changes will require flexibility and adaptation of beekeepers and their beekeeping practices alike, as spelled out previously and illustrated by the following quotes.

“As you know, bees are able to survive from the North of Europe to the Equator. They can deal with hot temperatures as long as they have water to ventilate. Honey bees as a species are not alarmed by climate change. But bees also need plants to survive. If crops, wildflowers, and vegetation are impacted, bees will be impacted too through their food resources.” –

*Scientist, 72 years old, France*

“Learning from the past, I would say that honey bees are very adaptive, flexible. Honey bees are kept in many different regions across the world. Europe alone has so many different regions and areas, and we have subspecies that are different and that have adapted differently. Of course, the challenge would be for new diseases. Honey bees can adapt and if they have a beekeeper that takes good care of them, new diseases should not be a problem that cannot be handled.” -

*Scientist, 38 years old, Germany*

### 3.2 Beekeeper survey

#### 3.2.1 Beekeeper characteristics

In total, 844 beekeepers completed the survey in 18 European countries. Eight countries had >50 beekeepers complete the survey, namely Belgium, the Netherlands, Germany, Portugal, Italy, Romania, and Finland. For analytical purposes, the countries were classified into four regions, based on the United Nations Geoscheme for Europe classification. A little more than half of the participants (53.9 %) resided in Western Europe, 18.5 % in Eastern Europe, 18.4 % in Southern Europe, and 9.2 % in Northern Europe. Participants' age ranged from 18 to 91 (mean age = 53 years), 80.7 % identified as male, and almost half of the participants (44.8 %) had been active as a beekeeper for longer than a decade. The size of beekeeping operations ranged from 1 to 6100 beehives, and around a fifth of the participants self-classified as being ‘rather professional’ or ‘fully professional’ based on the size of their beekeeping operation. Most beekeepers indicated keeping bees in a (mainly) rural area (89.0 %) and in a favourable natural environment (86.0 %). A favourable natural environment was defined as a score of 4 or higher (on a 1–5 scale) for at least one of the following three items referring to where the beehives are located, namely an area with forestry, an area with sufficient floral resources from early to late in the bee season, or an area with biodiverse floral resources.

Concerning output and performance variables, average honey production amounted to 17.2 kg/hive (S.D. = 13.9 kg/hive) among participating beekeepers who reported to have effectively produced honey in 2021 (93.0 % of the total sample). This is lower than the European average of 21 kg/hive based on data from 2017 and 2018, as estimated and reported in the National Apiculture Programmes 2020–2022 by the European Commission (2019). When explicitly asked how they evaluated 2021 compared to previous years from a honey production point of view, only 16.9 % and 4.8 % of the beekeepers indicated ‘good’ and ‘very good’ versus 25.4 % and 24.4 % who indicated ‘bad’ and ‘very bad’, respectively. Almost half of the beekeepers (48.2 %) indicated they were able to limit honey bee colony winter loss rates to <10 % on average during the previous five years. Finally, two thirds of the beekeepers had a pollination score of 15 or higher on a 4–20 scale. This score was calculated by aggregating the scores (on a 1–5 scale) of the beekeepers' self-reported contribution of their honey bees to agricultural, horticultural, and fruit production, and to the overall biodiversity of their environment through pollination.

#### 3.2.2 Perceived impacts of climate change

**Fig. 1:**
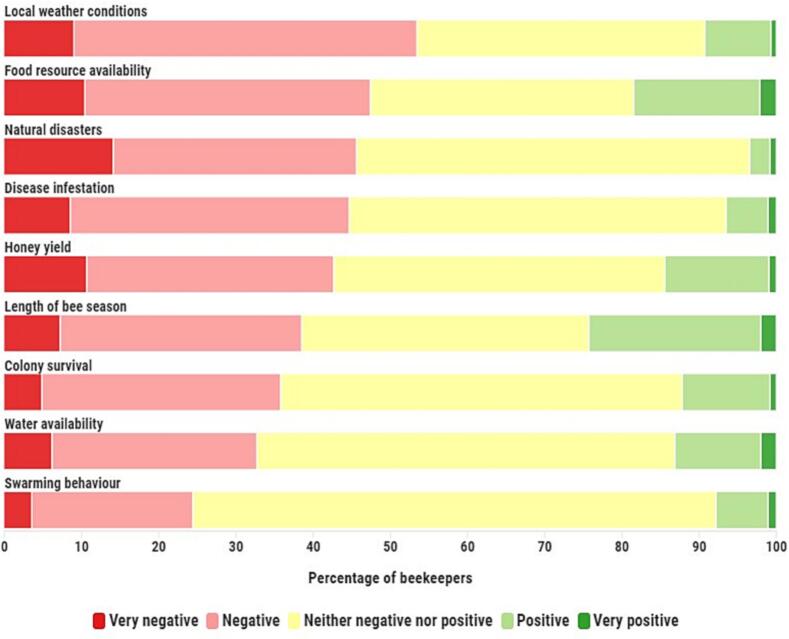
Perceived impacts of climate change on the natural environment, honeybee colony behaviour and performance (%, n=844).

Fig. 1 illustrates how beekeepers perceived the ways in which beekeeping has been affected by climate change, with local weather conditions, food resource availability, natural disasters, and disease infestation popping up as the most negatively perceived impacts. The large proportions of ‘neither negative nor positive’ answers in Fig. 1 may raise doubts around beekeepers' outspokenness on the various climate change-impacts. Yet these responses are more likely to reflect genuine mixed negative-positive or neutral perceptions rather than a systematic response bias since <3 % of the study sample systematically indicated this response option across all items. It is clear from our results that there are some impacts that were almost ubiquitously perceived as negative or neutral, such as the impact of natural disasters. However, there were also impacts that received more mixed responses, such as food resource availability and the changing length of the bee season. This is likely to be due to the large regional disparities in the way climate change is experienced, which was also a recurring theme in the stakeholder interviews.

**Fig. 2:**
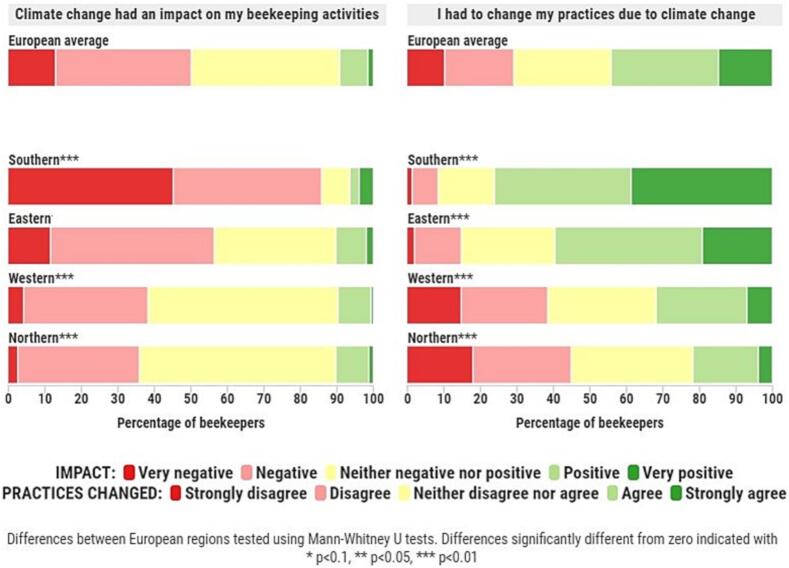
Regional disparity in the perceived impact of climate change and the need to adapt beekeeping practices due to climate change (%, n=844).

Indeed, beekeepers operating in Southern European regions reported significantly more negative effects – mostly citing worse food resource availability, lower honey yields and more adverse weather conditions. Whilst beekeepers in Northern European regions reported more positive effects – largely due to improved food resource availability and a more favourable length of the bee season. Fig. 2 confirms that the proportion of beekeepers that indicated they had been negatively or very negatively affected by climate change increases from north to south geographically and shows an almost identical increase in the number of beekeepers that agreed or strongly agreed that they had to change their practices in response to climate change. Furthermore, apart from the Eastern European region's response to the ‘perceived impact’ question, all responses significantly differed from the European averages pictured on top of Fig. 2.

Further bivariate analyses (Table 2, columns 2 and 3) point out that there are significant differences in terms of the perceived impact of climate change on beekeeping activities according to age and location, with climate change impacts being more negatively perceived by younger and rural beekeepers. Those that self-classified as professional, both in terms of size and skills, also had a significantly more negative perception of the impact of climate change on their beekeeping activities. Nevertheless, there were no differences based on the number of years active as a beekeeper, i.e., beekeepers who have been keeping bees for a longer period of time did not report a more negative perception of climate change's impacts than novices in beekeeping. Significant differences in the same variables could be observed in the responses to the question of whether beekeepers had to change their practices as a result of climate change as well. However, in this case there were also differences based on the number of years active as a beekeeper. New beekeepers (1–3 years active) indicated they had to change their practices significantly less than average, while those who had 11–25 years of experience reported that they had to adapt their practices significantly more.

**Table 2:** Personal and socioeconomic characteristics of the participating beekeepers (n = 844), mean (1–5) scores on questions probing for the perceived and experienced impact of climate change (CC), and percentage of participants that classified as ‘heavily impacted’ by climate change per group.

Characteristics	n	Mean		
		CC [negative-positive] impact on beekeeping activities⁎	CC forced me to change my beekeeping practices⁎	Percentage ‘heavily impacted’⁎⁎
Age group		***p*** ***<*** ***0.001***	***p*** ***<*** ***0.001***	***19.6 (p*** ***<*** ***0.001)***
35 or less	89	2.34	3.40	33.7 %
Between 36 and 64	568	2.42	3.27	35.4 %
65 or older	187	2.71	2.86	18.2 %
Gender		*p* *=* *0.169*	*p* *=* *0.150*	*0.19 (p* *=* *0.665)*
Male	681	2.50	3.22	31.7 %
Female	157	2.38	3.08	29.9 %
Other/non-disclosed	6	3.00	3.17	30.0 %
EU region		***p*** ***<*** ***0.001***	***p*** ***<*** ***0.001***	***152 (p*** ***<*** ***0.001)***
Northern	78	2.73	2.63	12.8 %
Western	455	2.68	2.86	18.7 %
Eastern	156	2.44	3.62	41.0 %
Southern	155	1.79	4.05	68.4 %
Education		*p* *=* *0.093*	***p*** ***=*** ***0.012***	***6.61 (p*** ***=*** ***0.037)***
Secondary or lower	267	2.42	3.38	37.1 %
Higher educ. – Ba level	244	2.45	3.11	30.7 %
Higher educ. – Ma level	333	2.54	3.11	27.3 %
Location		***p*** ***=*** ***0.010***	***p*** ***=*** ***0.012***	***8.35 (p*** ***=*** ***0.004)***
Rural	751	2.45	3.23	33.0 %
Urban	93	2.70	2.90	18.3 %
Professional in terms of size (self-reported)		***p*** ***<*** ***0.001***	***p*** ***<*** ***0.001***	***59.5 (p*** ***<*** ***0.001)***
Yes	160	2.23	3.89	56.9 %
No	684	2.54	3.04	25.4 %
Professional in terms of skills (self-reported)		***p*** ***<*** ***0.001***	***p*** ***<*** ***0.001***	***52.6 (p*** ***<*** ***0.001)***
Yes	300	2.32	3.60	47.0 %
No	544	2.56	2.97	22.8 %
Years active in beekeeping		*p* *=* *0.363*	***p*** ***<*** ***0.001***	***28.7 (p*** ***<*** ***0.001)***
1–3	144	2.48	2.83	18.8 %
4–10	322	2.48	3.11	27.3 %
11–25	198	2.39	3.52	43.9 %
26+	180	2.56	3.28	35.0 %
Operates in a favourable environment		*p* *=* *0.918*	*p* *=* *0.238*	*0.04 (p* *=* *0.839)*
Yes	726	2.48	3.17	31.3 %
No	118	2.48	3.33	32.2 %
Honey yield per hive		***p*** ***<*** ***0.001***	*p* *=* *0.232*	***9.81 (p*** ***=*** ***0.002)***
≤21 kg	561	2.41	3.23	34.7 %
>21 kg	209	2.67	3.12	23.0 %
Colony winter loss rate		*p* *=* *0.151*	*p* *=* *0.132*	***7.13 (p*** ***=*** ***0.028)***
0 %–10 %	407	2.53	3.14	27.0 %
10 %–20 %	259	2.46	3.17	34.7 %
>20 %	178	2.38	3.37	36.5 %
Pollination services score		***p*** ***=*** ***0.012***	***p*** ***<*** ***0.001***	***38.3 (p*** ***<*** ***0.001)***
≤14	302	2.53	2.90	24.2 %
15–16	206	2.53	3.04	28.0 %
>16	332	2.40	3.55	43.4 %

⁎ Group means compared using Mann-Whitney *U* tests for binary variables, and Kruskal-Wallis tests for categorical variables with more than two categories. Group means that significantly differ are denoted by *p* < 0.05.

⁎⁎ Association between the binary ‘heavily impacted’ variable and other categorical variables tested using chi-square tests of independence.

The variables ‘perceived impact of climate change on beekeeping activities’ and ‘having been forced to change beekeeping practices’ were significantly associated (chi-square = 313.30; *p* < 0.001). For example, 87.2 % of the beekeepers who perceived the impact of climate change to be ‘very negative’ indicated to have also been forced to change their beekeeping practices, while this proportion was only just over half for the beekeepers who perceived the impact of climate change to be ‘positive’ or ‘very positive.’ Beekeepers who classified as ‘heavily impacted’ by climate change, based on these two variables, accounted for 31.4 % (*n* = 265) of the total sample. Being classified as ‘heavily impacted’ was significantly associated with age, EU region, education, urban vs. rural location, the degree of professionalism (from pure hobby to fully professional), and the number of years active in beekeeping. Findings from these bivariate analyses (Table 2, column 4) suggest that the proportion of beekeepers stating heavy impacts from climate change increases with a rising degree of professionalism, an increasingly rural location of the beekeeping operation, a regional gradient from the Northern, over the Western and Eastern, to the Southern European regions, younger age, lower education, and more active years in beekeeping.

Importantly, beekeepers classifying as ‘heavily impacted’ by climate change associated significantly with the three key output and performance variables in beekeeping, namely honey yield, colony winter loss rate, and contribution through pollination. Honey-producing beekeepers who classified as ‘heavily impacted’ reported a significantly lower average honey yield per hive of 14.9 kg/hive, compared to 18.3 kg/hive for those who did not classify as ‘heavily impacted’ (Mann-Whitney *U* test, *p* = 0.007). The proportion of beekeepers reporting above-EU-average honey yields (i.e., >21.0 kg/hive) amounted 19.4 % among beekeepers classified as ‘heavily impacted’ vs. 30.0 % among those not classified as ‘heavily impacted’. Furthermore, beekeepers who classified as ‘heavily impacted’ reported significantly higher colony winter loss rates. One quarter (24.7 %) of the beekeepers classified as ‘heavily impacted’ by climate change reported an average colony winter loss rate over the past five years of 20 % or more, compared to about one fifth (19.5 %) of beekeepers who did not classify as ‘heavily impacted’. In a similar vein, the proportion of beekeepers classified as ‘heavily impacted’ is almost 10 % higher among beekeepers with a colony winter loss rate of 20 % or more compared to those with a winter loss rate lower than 10 %. Lastly, the average pollination score of beekeepers who classified as ‘heavily impacted’ was significantly higher than that of those who did not classify as ‘heavily impacted’ (Mann-Whitney *U* test, *p* < 0.001), respectively 16.3 vs. 14.9. Additionally, more than two fifths of the beekeepers with a pollination score higher than 16 classified as ‘heavily impacted’ by climate change.

#### 3.2.3 Multinomial logistic regression results

Table 3 reports the results of the multinomial logistic regression with the newly constructed binary variable ‘heavily impacted’ by climate change as the dependent variable. The coefficients of this multinomial logistic regression model with selected uncorrelated explanatory variables provide a clear indication of the specific contribution of a variable while simultaneously accounting for the impact of the other variables in the model. The regional disparities that were detected in the bivariate analyses are also significant predictors in the model. Compared to beekeepers in the Northern European regions, Eastern European beekeepers were 3.65 times more likely to classify as ‘heavily impacted’ by climate change, and for Southern European beekeepers this factor even amounted to being tenfold (Odds Ratio (OR) = 10.4). Moreover, the probability of being classified as ‘heavily impacted’ by climate change increased with almost one-third per unit increase of beekeepers' self-assessment of their degree of professionalism in terms of beekeeping skills (OR = 1.31), while it declined almost one-fifth as beekeepers had a more positive report of 2021 in terms of the economic performance of their beekeeping operation (OR = 0.81). Finally, the surroundings of a beekeeper's operation turned out to be very important: the degree to which beekeepers indicated that their hives are surrounded by forests (OR = 1.34) was a positive predictor of the probability of being classified as ‘heavily impacted’ by climate change, whereas hives being surrounded by sufficient floral resources for the entire length of the bee season (OR = 0.78) and contentedness with policy measures addressing environmental issues in their beekeeping environment (OR = 0.76) were negative predictors, i.e., a stronger presence of these factors lowered the probability of being classified as ‘heavily impacted’ by climate change.

**Table 3:** Results of the multinomial logistic regression identifying determinants of beekeepers classifying as ‘heavily impacted’ by climate change (*n* = 265) or not (*n* = 579).

Variables		β	SE	p	OR (95 % CI)
EU region	Northern	(Base level)			
	Western	0.52	0.38	0.167	1.69 (0.80–3.54)
	Eastern	1.29	0.40	0.001	3.65 (1.68–7.93)
	Southern	2.34	0.40	<0.001	10.4 (4.73–22.7)
Professionalism (based on skills)		0.27	0.07	<0.001	1.31 (1.15–1.49)
Years active in beekeeping		0.02	0.01	0.018	1.02 (1.00–1.03)
Perception of 2021 from economic point of view		−0.21	0.09	0.016	0.81 (0.69–0.96)
Colonies are surrounded by forests		0.29	0.08	<0.001	1.34 (1.15–1.56)
Colonies are surrounded by sufficient floral resources		−0.25	0.09	0.004	0.78 (0.66–0.92)
Policy measures address environmental issues		−0.27	0.09	0.002	0.76 (0.64–0.90)

Goodness of fit: pseudo-R^2^ = 0.212, percentage of correct predictions is 76.5 % compared to 68.6 % for the naïve model.

**Fig. 3:**
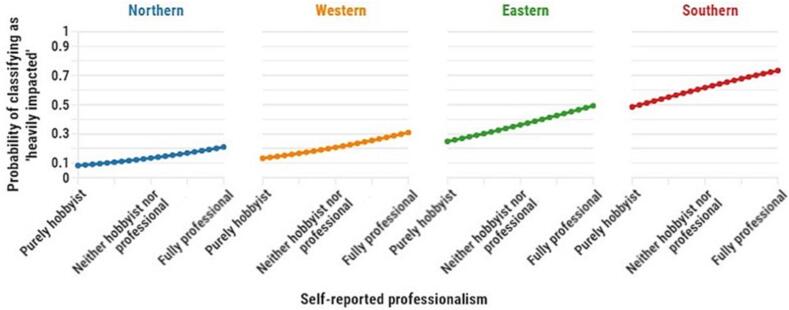
Simulated probability of classifying as ’heavily impacted’ by climate change from purely hobbyist to fully professional beekeepers per EU region.

Finally, the probability of classifying as ‘heavily impacted’ by climate change has been simulated using the coefficient estimates reported in Table 3 across beekeepers' degree of professionalism and for different EU regions while keeping all other variables in the model at their sample mean. As a result, Fig. 3 depicts how the probability of classifying as ‘heavily impacted’ by climate change increases as beekeepers are more professional in terms of skills, but it also once more very clearly illustrates the north-south gradient in the perceived impact of climate change. This simulation illustrates that the probability of being classified as ‘heavily impacted’ by climate change ranges from <10 % to just above 20 % for a hobbyist and a professional beekeeper, respectively, in Northern Europe, while the equivalent range of this probability amounts from 50 % for a hobbyist beekeeper to >75 % for a professional beekeeper in Southern Europe.

## 4 Discussion and conclusion

### 4.1 Main findings discussed

During the in-depth interviews, expert stakeholders identified a diverse – albeit interlinked – set of climate change impacts on the beekeeping sector and its activities. The beekeeper survey, and its findings have provided an indication and gradients (e.g., regional differences) for which climate change impacts are most pressing, such as irregular local weather conditions, varying food resource availability, the occurrence of natural disasters, and more intense pest and disease infestation. These findings are generally in line with those of the studies by Flores et al. (2019), Medina-Cuéllar et al. (2018), Newman et al. (2021), Novelli et al. (2021), and Vercelli et al. (2021), which reported the same urgent climate change-related impacts among beekeepers in specific countries such as Spain, Mexico, Kenya, and Italy.

Interestingly, stakeholder views and opinions gained in the interviews on perceived and expected impacts of climate change were mostly negative, with some positive aspects mentioned, but they expected both negative and positive impacts to vary across European regions. This view was confirmed by the analysis of the beekeeper survey data, where negatively perceived impact scores outweighed positive scores, although positive sentiments were not totally absent. The analyses reveal there may be potential ‘winners’ and ‘losers’ among European beekeepers. Certainly, many beekeepers consider they have been negatively impacted, with half of the participating beekeepers in the survey (*n* = 423, 50.1 %) indicating being (very) negatively affected by climate change. However, this proportion amounted to 85.8 % in Southern European regions, confirming the vulnerability of the Mediterranean to global warming (Flores et al., 2019). The survey data clearly illustrates that the perceived impacts of climate change and the need to adapt beekeeping practices become progressively more negative and pressing, respectively, along the North-South gradient in Europe.

Moreover, on the one hand, stakeholders were not overly pessimistic while referring to the resilience of honey bees as a species, and to their flexibility and ability to adapt to changing circumstances and environments. As noted by Le Conte and Navajas, “[b]ees of the *Apis* genus are distributed throughout the world in highly diverse climates. The *Apis mellifera* species' […] distribution range extends to sub-Saharan Africa, northern Europe, and Central Asia” (Le Conte and Navajas, 2008, p.1). Stakeholders did note that, while *Apis mellifera* as a species are resilient, there might be underlying shifts in the dominance of subspecies as a result of climate change. For example, the *Apis mellifera sahariensis –* currently inhabiting oases in the Sahara Desert – could become more widespread as it is adapted to extreme heat (Le Conte and Navajas, 2008). On the other hand, stakeholders underscored the challenge for beekeepers to demonstrate a similar degree of adaptivity and flexibility in their beekeeping management practices; a sentiment shared by many other studies (Decourtye et al., 2019; Flores et al., 2019; Kouchner et al., 2019; Novelli et al., 2021; Steinhauer et al., 2021; Vercelli et al., 2021). The beekeeper survey data confirmed that a substantial share of beekeepers (44.2 %) have already changed their beekeeping practices. This is particularly the case for beekeepers in specific regions, notably Southern (76.1 %) and Eastern Europe (59.6 %). The proportion of Southern beekeepers that indicated they had already changed their beekeeping practices is in line with results from studies in Chile (Gajardo-Rojas et al., 2022) and Mexico (Gallardo-López et al., 2021), which reported that 80.5 % and 80.7 % of beekeepers had been forced to adapt their practices due to climate change, respectively. Some possible examples of strategies beekeepers could adopt to cope with climate change include providing supplemental feed resources (e.g., syrups or sugar) as indicated in the quote below, and a more intense application of transhumance (Novelli et al., 2021; Vercelli et al., 2021).

“There will be so much more work for beekeepers, and it is not cheap for the beekeepers to be completely aware of their bees' [wellbeing]. They must also give them some additional feed, not just for the winter, but even for surviving in the late summer. And this is just one of the things that are changing when we look at the global climate crisis.” –

*Representative of a beekeeping association, 42 years old, Slovenia*

Being classified as ‘heavily affected’ by climate change – a binary variable that was constructed by grouping those beekeepers that simultaneously indicated they felt (very) negatively affected by climate change and (strongly) agreed they had to adapt their beekeeping practices – was associated with lower honey yields, higher colony winter loss rates, and a bigger self-reported contribution to food production and biodiversity through their honey bees' pollination services. Our findings herewith suggest that climate change for the European apicultural sector has direct and detrimental impacts on honey production and honey bee colony survival, as well as indirect impacts on crop production and biodiversity through pollination. The fact that these effects are more prominent in Southern European countries is not surprising, for example, Medina-Cuéllar et al. (2018) estimated that a 1 % increase in temperature decreases honey production by 0.13 %, while Patel et al. (2020) and Switanek et al. (2017) reported that winter mortality increases if the local weather conditions were drier and hotter in the preceding year. The strong regional gradient found in this research highlights the need for future pan-European research. Especially the effects of climate change in more Northern European regions might be of interest, as it has been projected to become a region with low climate stability (Hlásny et al., 2021).

To identify factors that determine the likelihood of beekeepers classified as ‘heavily impacted’ by climate change, this paper presented the results of a multinomial regression analysis with the ‘heavily impacted’-binary variable as the dependent variable. The results provided evidence that negative impacts of climate change are particularly strong in Southern Europe and more intensely experienced by professional beekeepers. A potential explanation for the observed differences between hobbyists and professionals, is that professional beekeepers may regard that there are fewer possibilities for adaptation, since they are already operating at a maximum capacity, at a certain optimum or limit, or with a minimal margin. Additionally, professional beekeepers may be more alert to warning signals in their colonies, and more attuned to climate change impacts and the necessity to adapt even if they face operational constraints. By contrast, hobbyists – who are not dependent on beekeeping as their main or sole source of income – can more easily scale up or down, or even drastically change their beekeeping management practices, even though this might go hand in hand with an extended period of lower (economic) performance or honey yields. Similarly, our results show the likelihood of being classified as ‘heavily impacted’ by climate change increases with an increasing number of years active in beekeeping. It is less likely that novices or starters experience negative impacts or already had to change their beekeeping practices (since only recently started) than experienced beekeepers who have been active for up to several decades.

Furthermore, the findings confirmed the importance of suitable landscapes for beekeeping when facing challenges as a result of climate change. Specifically, the crucial role of the so-called ‘flowering arch’ or ‘blooming bridge’ [literally translated from Dutch ‘bloeiboog’]. This composition of diverse plants and trees with different flowering periods from early on till late in the bee season (Naturalis, 2022), is an important aspect of floral resources in the vicinity of apiaries. However, it is acknowledged that, because of climate changes, the ‘flowering arch’ may have become stretched, less predictable and/or less well synchronised with developments and foraging activities of honey bee colonies. Contrary to initial expectations, the analysis indicated that beekeepers with hives located in forested environments experience a higher likelihood of being classified as ‘heavily impacted’ by climate change.

Forests are typically regarded as providing a favourable beekeeping environment, e.g., honey production increases 0.05 % with every 1 % increase of forest area according to Medina-Cuéllar et al. (2018). The finding that beekeepers with apiaries near/in forests have a higher probability of classifying as ‘heavily impacted’ could mean that, when their normally favourable environment suddenly alters due to climate change, these beekeepers feel more intensely affected as opposed to beekeepers with hives located in less optimal environments, whose climate change related losses are felt less intensely. Indeed, Buchori et al. (2022) reported that awareness of adverse climate effects as a cause of bee mortality was especially prevalent among beekeepers who keep their hives in forests rather than agricultural fields or gardens. Another possible explanation for this finding could be increased competition with wild, forest-dwelling pollinator species as a result of declining food resource availability. A study by Markwell et al. (1993), e.g., found that aggression would occur between bees and wasps feeding on honeydew from beech trees once food resources run out. Unfortunately, forests are affected harshly by climate change through temperature stress or through natural disasters such as droughts, fires, floods, storms, and landslides (FAO, 2015). This – at first sight unexpected, yet plausible – outcome of our analysis may be of interest for future research, such as exploring whether landscape context might overshadow effects of climate change on honey bees, or eventual increased competition between honey bees and wild pollinators which inhabit forests driven by climate change.

“Almost 80 percent of Austrian honey comes from honeydew from coniferous trees, but if the climate becomes hotter, the conifers will move further up into the higher Alpine regions. If these forests disappear due to climate change, we will lose our main honey dew source and honey producing trees in the lowlands, which is really a big problem.” –

*Apiary product quality inspector, 60 years old, Austria*

Last but not least, from a policy perspective, it is important that the reported presence of policies that address environmental issues added favourably to being less likely to be classified as ‘heavily impacted’ by climate change. Although these findings do not allow to conclude anything about the eventual effectiveness of such policies, the results at least suggest that dedicated policies convey trust and may entail the potential to address climate change-related challenges facing beekeepers and the beekeeping sector in Europe. Based on this study's findings, the focus of such policies should be on mitigating the potential adverse impacts of local weather conditions and natural disasters, and on preserving or shaping optimal environmental conditions for the provision of sufficient floral resources to ensure future food resource availability for honey bees and pollinators more generally. These policies need to differentiate between European regions and beekeeper types ranging from hobbyists to professionals, depending on the felt impacts of climate change, and to devote particular attention to role of forestry environments.

### 4.2 Limitations

It should be acknowledged that this study faces some limitations stemming from the applied participant recruitment and data collection procedure, which were online and implying that participation was based on self-selection. As a result, the survey sample might be biased towards beekeepers with some degree of ICT-literacy and a strong involvement in the research topic. First, the use of a non-probability sampling method imposes limits on the representativeness of the study sample for the overall population of European beekeepers, as well as on the generalisation of study findings beyond the characteristics of the study sample. Achieving representativeness is an issue of concern in many studies involving populations from whom no sampling frame and population statistics are available such as beekeepers on a national or regional level across Europe. This situation prevents the use of probability random sampling methods and ultimately also assessing sample representativeness. As with the COLOSS beekeeper surveys, we followed the strategy to aim for as many answers from beekeepers as possible through using multiple routes of participant recruitment (Brodschneider et al., 2018). An advantage of the approach we followed is that the same data collection method has been used in each of the countries where data have been collected. It should nevertheless be recognised that some important European beekeeping countries are poorly or not at all represented in our study sample, e.g., Spain, Czech Republic, Greece, and Denmark. We did approach numerous national beekeeping associations, however some declined to participate and distribute our survey link among their members in order to avoid ‘over-surveying’ their beekeepers, as well as possible conflicts with their own surveying efforts - decisions we respected. Because of differences in response rates across countries and eventual representativeness, we opted not to analyse the data at country level but to group responses into EU-regions for further analysis.

A second issue of concern is non-response and selection bias given the self-selection nature of our sample. To avoid this type of bias, both the qualitative exploratory study with stakeholders and the quantitative descriptive study with beekeepers were (rightfully) introduced as a study on the socioeconomics of beekeeping in the context of European research aiming to pave the way to healthy and sustainable beekeeping in Europe. Specific questions about climate change were embedded in interview and survey sections focusing on environmental quality and its potential impact on beekeeping. It is therefore unlikely that stakeholders and beekeepers with strongly outspoken views on the impact of climate change are either over- or underrepresented in the study sample.

Third, the collected data are based on self-reports and self-assessments, which may be prone to social desirability bias. The latter warrants caution especially in the treatment and interpretation of single variables, e.g., the mere proportion of beekeepers reporting that climate change had a (very) negative impact on their beekeeping activities. Efforts have been made to address limitations resulting from the collection of self-reported data through multistage questionnaire pilot-testing, the use of multiple-item rather than singe-item measures whenever possible, randomisation of question items within questions and of questions within survey sections and guaranteeing anonymous and aggregated data analysis and reporting.

### 4.3 Conclusions

Notwithstanding the abovementioned limitations, this study into the perceived and experienced impact of climate change on beekeeping in Europe provides novel and original insights. Our findings are a result of this study's mixed methods approach including both stakeholders and beekeepers, its pan-European coverage, and the implementation of multiple and complimentary qualitative exploratory and quantitative conclusive data analysis methods. In-depth interviews with stakeholders from diverse parts of the apicultural sector uncovered four general observations: 1) climate change has/will have various, interlinked impacts on beekeeping; 2) these impacts are predominantly negative but not exclusively, hence some beekeepers may ‘win’, some may ‘lose’; 3) (honey)bees as a species are deemed to be resilient and therefore believed to be able to cope with the challenges imposed by climate change, at least if properly managed; 4) climate change is perceived as a main challenge for beekeepers who will need to demonstrate a certain degree of flexibility, adaptability and resilience alike. Results from the large-scale beekeeper survey that was informed by these interviews confirmed the predominantly negative nature of climate change impacts, and clearly highlighted regional differences, with Southern European beekeepers being the most negatively affected and Northern European beekeepers experiencing more positive effects. The likelihood of beekeepers being classified as ‘heavily impacted’ by climate change is not only determined by their geographical location, but also by their self-reported degree of hobby-ism versus professionalism, and number of years active in beekeeping. Beekeepers whose hives are surrounded by sufficient floral resources feel less heavily impacted, while the opposite holds for beekeepers whose hives are located in forested areas. Finally, local policies addressing climate change facing the apicultural sector emerged as decreasing the beekeepers' likelihood of feeling heavily impacted by climate change, herewith underscoring the relevance of such policies to foster healthy and sustainable beekeeping in Europe.

The following is the supplementary data related to this article.

Supplementary material Question formulation, measurement scale and response options for the dependent variables in this study. Supplementary material

## CRediT authorship contribution statement

MVE: Data curation, Methodology, Formal analysis, Visualisation, Writing – original draft; JHW: Conceptualisation, Methodology, Investigation, Writing – review & editing; FA: Conceptualisation, Methodology, Investigation, Writing – review & editing; YH: Supervision, Writing – review & editing; DCDG: Resources, Supervision, Writing – review & editing; WV: Resources, Conceptualisation, Methodology, Investigation, Supervision, Writing – original draft.

## Declaration of competing interest

The authors declare that they have no known competing financial interests or personal relationships that could have appeared to influence the work reported in this paper.

## Data Availability

Data will be made available on request.
