# Supplementary material for: Beekeeping in Europe facing climate change: A mixed methods study on perceived impacts and the need to adapt according to stakeholders and beekeepers
Source: Sci Total Environ. 2023 Aug 25;888:164255. doi: 10.1016/j.scitotenv.2023.164255 (PMC10280316; doi:10.1016/j.scitotenv.2023.164255)
Supplement: Supplementary material — Question formulation, measurement scale and response options for the dependent variables in this study. [file mmc1.docx]

Beekeeping in Europe facing climate change: a study on perceived impacts and the need to adapt according to stakeholders and beekeepers

**Supplementary material**: Question formulation, measurement scale and response options for the dependent variables in this study

| Question formulation | Measurement scale | Response options |
| --- | --- | --- |
| Question 1:  Please indicate to what extent you agree or disagree with the following statement?  “Climate change has forced me to change my beekeeping practices.”* | 5-point interval | 1 = Strongly disagree  2 = Disagree  3 = Neither agree nor disagree  4 = Agree  5 = Strongly agree |
| Question 2:  “According to my personal experience, climate change has a … impact on my beekeeping activities.”* | 5-point interval | 1 = Very negative  2 = Negative  3 = Neither negative nor positive  4 = Positive  5 = Very positive |
| Question 3:  Please indicate to what extent you believe climate change has a positive or negative impact on your beekeeping activities, based on your personal experience?  *[For each of the following items, which were presented in randomised order to the survey participants]*  - Food resource availability  - Water availability  - Local weather conditions  - Natural disasters like fires or floodings  - Length of the bee season  - Disease infestation  - Honey yield  - Colony survival  - Swarming behaviour | 5-point interval | 1 = Very negative  2 = Negative  3 = Neither negative nor positive  4 = Positive  5 = Very positive |

*: Beekeepers who scored ‘Strongly agree’ or ‘Agree’ on question 1, and ‘Very negative’ or ‘Negative’ on question 2 were classified as ‘heavily impacted’ by climate change.
